# Safety and Efficacy of High-Dose Folinic Acid in Children with Autism: The Impact of Folate Metabolism Gene Polymorphisms

**DOI:** 10.3390/nu17091602

**Published:** 2025-05-07

**Authors:** Caiyun Zhang, Yanlin Chen, Fang Hou, Yanzhi Li, Wanxin Wang, Lan Guo, Caixia Zhang, Li Li, Ciyong Lu

**Affiliations:** 1 Department of Medical Statistics and Epidemiology, School of Public Health, Sun Yat-sen University, Guangzhou 510275, China; zhangcy28@mail2.sysu.edu.cn (C.Z.); liyzh96@mail2.sysu.edu.cn (Y.L.); wangwx65@mail.sysu.edu.cn (W.W.); guolan3@mail.sysu.edu.cn (L.G.); zhangcx3@mail.sysu.edu.cn (C.Z.); 2 Guangdong Provincial Key Laboratory of Food, Nutrition and Health, Sun Yat-sen University, Guangzhou 510275, China; 3 Department of Pediatric Neurorehabilitation, Maternity and Children Health Care Hospital of Luohu District, Shenzhen 518019, China; ningxinyilin@126.com (Y.C.); houfang@alumni.hust.edu.cn (F.H.)

**Keywords:** autism, folinic acid, safety, efficacy, folate metabolism gene polymorphisms

## Abstract

**Background/Objectives:** Research on the safety and efficacy of high-dose folinic acid in Chinese children with autism spectrum disorder (ASD) is limited, and the impact of folate metabolism gene polymorphisms on its efficacy remains unclear. This trial aimed to evaluate the safety and efficacy of high-dose folinic acid intervention in Chinese children with ASD and explore the association between folate metabolism gene polymorphisms and efficacy. **Methods:** A 12-week randomized clinical trial was conducted, including 80 eligible children with ASD, randomly assigned to an intervention group (*n* = 50) or a control group (*n* = 30). The intervention group was administered folinic acid (2 mg/kg/day, max 50 mg/day) in two divided doses. Efficacy was measured using the Psycho-Educational Profile, Third Edition (PEP-3) at baseline and 12 weeks by two trained professionals blind to the group assignments. Methylenetetrahydrofolate reductase (*MTHFR* C677T, *MTHFR* A1298C), methionine synthase (*MTR* A2756G), and methionine synthase reductase (*MTRR* A66G) were genotyped by the gold standard methods in the intervention group. **Results:** 49 participants in the intervention group and 27 in the control group completed this trial. Both groups showed improvements from baseline to 12 weeks across most outcome measures. The intervention group demonstrated significantly greater improvements in social reciprocity compared to the control group. Children with *MTHFR* A1298C or *MTRR* A66G mutations demonstrated greater improvements in various developmental domains than wild type. Folinic acid may be more effective in certain genotype combinations, such as *MTHFR* C677T and A1298C. No significant adverse effects were observed during the intervention. **Conclusions:** High-dose folinic acid may be a promising intervention for children with ASD, and its efficacy is associated with folate metabolism gene polymorphisms. High-dose folinic acid intervention may promote better neurodevelopmental outcomes by alleviating folate metabolism abnormalities caused by single or combined mutations in folate metabolism genes.

## 1. Introduction

Autism spectrum disorder (ASD) is a complex neurodevelopmental disorder characterized by impairments in communication and social interaction, restricted interests, and repetitive and stereotyped behaviors [1]. The prevalence of ASD is rising worldwide, with approximately 1 in 54 children suffering from this disorder [2,3]. Individuals with ASD require lifelong continuous medical and social support, which places a heavy burden on affected families and the healthcare system [4,5]. Despite extensive research, there are still no approved drugs to treat the core symptoms or underlying pathophysiological abnormalities of ASD [6]. Therefore, there is an urgent need to explore effective interventions targeting underlying biochemical abnormalities of ASD.

Numerous studies have demonstrated that one of the most promising treatable pathophysiological targets is folate metabolism abnormalities for ASD [6,7]. Normal folate metabolism is essential for one-carbon metabolism and normal nervous system development [7,8]. Folinic acid (also known as leucovorin), a reduced form of folate, has recently gained attention as a potential pharmacological intervention for children with ASD [9,10]. Folinic acid can directly enter the folate cycle without requiring reduction by dihydrofolate reductase, thereby effectively normalizing folate-dependent one-carbon metabolism, correcting abnormalities in methylation and transsulfuration pathways, and alleviating oxidative stress and inflammatory responses [11,12,13]. Several clinical trials have shown that high-dose folinic acid supplementation can alleviate autistic traits, particularly in language expression, communication, and social interaction [11,14,15,16]. Although folinic acid intervention shows potential as a therapeutic strategy to alleviate autistic traits, most studies to date have focused on Western populations, with further studies warranted to address this gap in Chinese children with ASD [9].

Notably, the efficacy of folinic acid intervention may be influenced by folate metabolism gene polymorphisms related to ASD [17,18]. Methylenetetrahydrofolate reductase (*MTHFR*) C677T and A1298C, methionine synthase (*MTR*) A2756G, and methionine synthase reductase (*MTRR*) A66G are the most frequently studied polymorphisms in folate metabolism pathways and have been shown to affect enzymatic activity and normal folate metabolism [9,11,12,17,19,20]. Reduced MTHFR activity leads to impaired conversion of 5,10-methylenetetrahydrofolate to 5-methyltetrahydrofolate, the biologically active form of folate, thereby decreasing the pool of bioavailable folate in the body [21]. Similarly, reduced activity of MTR and MTRR may hamper the remethylation of homocysteine to methionine, resulting in the accumulation of homocysteine and the reduction of S-adenosylmethionine, which is a critical methyl donor for DNA methylation and other cellular methylation reactions [21]. High-dose folinic acid supplementation can provide sufficient substrate for the folate cycle, which can improve folate metabolism abnormalities caused by reduced activity of MTHFR, MTR, or MTRR [22,23,24]. Moreover, studies have shown that the phenotypic effects of the *MTHFR* C677T and A1298C polymorphisms are influenced by folate levels [24,25]. Maternal folic acid supplementation during pregnancy is associated with a reduced risk of ASD in offspring carrying the *MTHFR* C677T mutation [23,26]. However, there is a limited study to explore the association between folate metabolism gene polymorphisms and the efficacy of folinic acid intervention. Understanding the association between folate metabolism gene polymorphisms and efficacy is crucial for optimizing folate therapy in ASD and identifying the subgroups most likely to benefit.

Therefore, this 12-week randomized clinical trial aimed to (1) evaluate the safety and efficacy of high-dose folinic acid intervention (2 mg/kg/day, maximum 50 mg/day) in Chinese children with ASD; and (2) explore the association between folate metabolism gene polymorphisms (*MTHFR* C677T, *MTHFR* A1298C, *MTR* A2756G, and *MTRR* A66G) and efficacy. This study helps to elucidate the potential benefits of folinic acid supplementation on developmental outcomes in children with autism and provides an important genetic basis for personalized nutritional interventions, which contribute to the development of precision medicine strategies for this population.

## 2. Materials and Methods

### 2.1. Participants and Study Design

**Figure 1:**
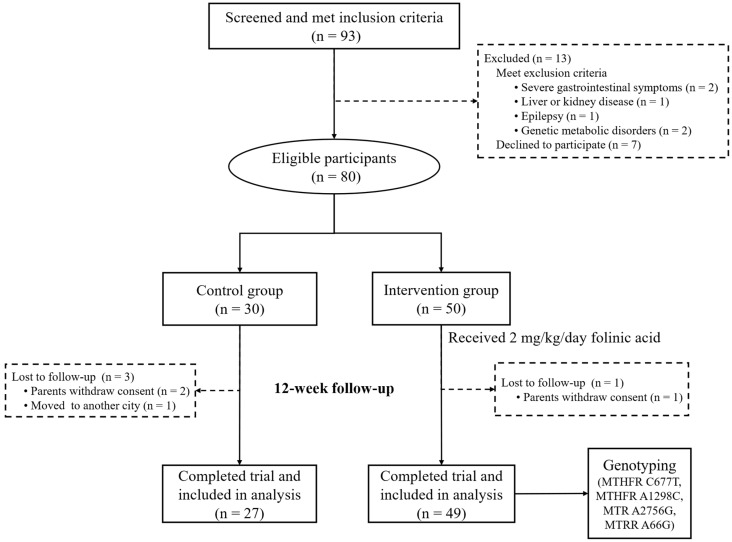
Flowchart of this study.

This randomized clinical trial was conducted from August 2021 to August 2023 at the Maternity and Children Health Care Hospital of Luohu District, Shenzhen, China. Eighty eligible participants were randomly assigned to either the intervention (*n* = 50) or the control group (*n* = 30). Efficacy assessments were performed at baseline and at the end of the 12-week intervention. The inclusion criteria were children aged 3 to 6 years diagnosed with ASD by two specialized clinicians according to the criteria outlined in the fifth edition of the Diagnostic and Statistical Manual of Mental Disorders (DSM-5). Exclusion criteria included the presence of severe gastrointestinal symptoms, congenital heart disease, liver or kidney disease, epilepsy, brain malformations, taking antipsychotic medications, and previous or current use of folate-related drugs or any medications known to affect folate metabolism (e.g., vitamin or mineral supplements). Additionally, participants with mitochondrial diseases, other genetic metabolic disorders, and severe mental or neurological conditions were also excluded. Informed consent was obtained from all participants’ legal guardians before enrollment. Four participants were lost to follow-up during the study period, including three in the control group (two due to parental withdrawal of consent and one due to relocation) and one in the intervention group (due to parental withdrawal of consent). The detailed flowchart of this study is presented in Figure 1.

### 2.2. Interventions

Participants in the intervention group were administered folinic acid (Guangdong Lingnan Pharmaceutical Co., Ltd., Shaoguan, China) at a dose of 2 mg/kg per day (maximum of 50 mg per day), given orally in two divided doses with an interval of 6 to 8 h, for 12 weeks. The dosage of folinic acid was based on previously published double-blind, placebo-controlled randomized clinical trials that demonstrated both the efficacy and safety in children with ASD [11,14]. Folinic acid doses greater than 5 mg/day are generally classified as high-dose [27]. Folinic acid can be swallowed directly or crushed and mixed with food or drink for ease of consumption. Adherence to the intervention was monitored by collecting feedback from parents and conducting pill counts throughout the study.

In addition, both the intervention and control groups received a 12-week comprehensive rehabilitation education intervention, which was grounded in the Treatment and Education of Autistic and Related Communication Handicapped Children (TEACCH) framework. TEACCH constitutes a multidimensional therapeutic and educational program designed for children with ASD, utilizing individualized and structured teaching strategies to enhance cognitive functioning, social communication skills, and adaptive behaviors [28]. Implemented through a half-day classroom-based training model, the protocol integrated seven core modules: (1) structured teaching, (2) music therapy, (3) art-based activities, (4) language and cognitive training, (5) interactive social games, (6) daily living skills development, and (7) sensory integration therapy.

### 2.3. Outcome Measures

This study evaluated the efficacy of folinic acid intervention using the Psycho-Educational Profile, Third Edition (PEP-3). PEP-3 is a mature tool for evaluating intervention efficacy in ASD and can more accurately and comprehensively assess and monitor the development of children with ASD aged 6 months to 7 years [29,30]. The PEP-3 comprises 172 items organized into 10 subtests, which include three composites for communication ability, three for motor ability, and four for maladaptive behaviors [29]. The evaluation was performed by two independent professionals who were not involved in the study and blind to the group assignments but had specialized training and experience in administering PEP-3. In this assessment, lower raw scores indicate a higher level of autistic traits. The Cronbach’s alpha coefficients for the PEP-3 at baseline and after the 12-week intervention were 0.96 and 0.97, respectively.

### 2.4. Adverse Effects

The Treatment Emergent Symptom Scale (TESS) was used to monitor the adverse effects of high-dose folinic acid supplementation [31]. Participants were withdrawn from the study if their guardians withdrew consent or if they experienced adverse reactions during the intervention, such as difficulty sleeping, insomnia, hyperactivity, agitation, or abnormal neurological reflexes.

### 2.5. Genotyping

Genotyping was performed using polymerase chain reaction (PCR) and Sanger sequencing for *MTHFR* C677T (A222V, rs1801133), *MTHFR* A1298C (E429A, rs1801131), *MTR* A2756G (D919G, rs1805087), and *MTRR* A66G (I22M, rs1801394) in the intervention group [19,32]. This approach is considered the gold standard due to its high reliability and accuracy [33]. Peripheral blood samples were collected from participants at baseline, and genomic DNA was extracted using the Magnetic Universal Genomic DNA Kit (DP705, Tiangen Biochemical Technology Co., Ltd., Beijing, China). Primers were designed using Primer Premier 5.0 and synthesized by Shanghai MAP Biotech Co., Ltd. (Shanghai, China). The PCR amplification reaction system and PCR procedure settings are provided in the Supplementary Materials, Tables S1 and S2. The PCR products were purified using the E.Z.N.A. Gel Extraction Kit (D2500-01, Omega Bio-Tek, Norcross, GA, USA) according to the manufacturer’s instructions. Sequencing was conducted on an ABI 3730XL sequencer using fluorescently labeled dideoxynucleotides to generate chromatograms, which were analyzed to determine the genotype. The primer sequences and PCR product lengths for genotyping folate metabolism genes, along with a summary of the genotyping results, are provided in the Supplementary Materials, Tables S3 and S4. The locations of the *MTHFR*, *MTR*, and *MTRR* genes in the folate metabolism pathway are shown in Figure S1.

### 2.6. Statistical Analysis

Descriptive statistics were used to summarize the baseline demographic and clinical characteristics of the ASD subjects. Continuous variables were summarized as means with standard deviations (SD), and group differences were assessed using the *t*-test. Categorical variables were reported as frequencies and percentages, and between-group comparisons were assessed using Fisher’s exact test due to small sample sizes in certain subgroups. Hardy–Weinberg equilibrium of genotype frequencies of different genes was assessed using the χ^2^ test [21].

Independent sample *t*-tests were used to evaluate the efficacy of high-dose folinic acid intervention in Chinese children with ASD [34]. Linear mixed-effects models were performed to examine the relationship between independent gene polymorphisms (*MTHFR* C677T, *MTHFR* A1298C, *MTR* A2756G, and *MTRR* A66G) and changes in the outcome variables (PEP-3 scores) over time [35]. A separate model was fitted for each outcome variable, reporting the polymorphism–time interaction effect, 95% confidence intervals (CI), and *p*-values for each. Moreover, linear regression models were used to explore the association between the combination of different genotypes and the efficacy of folinic acid intervention. All analyses were adjusted for baseline scores of each outcome variable, age at enrollment, ethnicity, preterm birth, birth weight, mode of delivery, parental ages at birth, parental education levels, and the scores of the Autism Behavior Checklist (ABC) and Childhood Autism Rating Scale (CARS). Analyses were conducted using R (version 4.4.1), and *p*-values less than 0.05 were considered statistically significant.

## 3. Results

### 3.1. Baseline Characteristics of Children with ASD

As shown in Table 1, 49 participants in the intervention group and 27 participants in the control group completed the trial and were included in the final analysis. The mean age at enrollment was comparable between control and intervention groups (51.30 ± 12.94 months vs. 51.27 ± 9.75 months; *p* = 0.991). There were significant differences in birth weight (*p* = 0.041) and maternal education level (*p* = 0.027) between the control and intervention groups. There were no significant differences between intervention and control groups in terms of gender, ethnicity, preterm birth, mode of delivery, paternal and maternal ages at birth, father’s education levels, ABC and CARS scores (all *p* > 0.05).

### 3.2. Safety and Efficacy of High-Dose Folinic Acid Intervention in Chinese Children with ASD

Table 2 compares the baseline and 12-week scores, as well as the score changes between the intervention and control groups across various subtests and composite measures. Overall, both groups showed improvements across most measures from baseline to 12 weeks within each group. The intervention group showed a significant advantage over the control group in the social reciprocity subtest. This subtest exhibited a statistically significant between-group difference, with the intervention group showing a greater mean change of 1.53 (SD = 1.98) compared to 0.33 (SD = 1.71) in the control group (mean difference = 1.20, 95% CI: 0.29, 2.10; *p* = 0.010). Additionally, the high-dose folinic acid supplementation in this trial was well tolerated among children with ASD, with no significant adverse reactions reported during the observation period.

### 3.3. Association of Single Folate Metabolism Gene Polymorphism with the Efficacy of Folinic Acid

The distribution of the four polymorphism genotypes was consistent with Hardy-Weinberg gene equilibrium (Supplementary Materials, Table S5). There were no significant sex differences in the distribution of the gene polymorphisms *MTHFR* A1298C, *MTHFR* C677T, *MTR* A2756G, and *MTRR* A66G (all *p* > 0.05) in the intervention group (Supplementary Materials, Table S6). At baseline, children with ASD carrying the *MTHFR* A1298C (AC/CC) variant had lower social reciprocity scores, and those with the *MTR* A2756G (AG) variant had lower gross motor and composite motor scores compared to the wild type, while no significant differences were found between the wild type and variant for the other loci (Supplementary Materials, Table S7).

**Figure 2:**
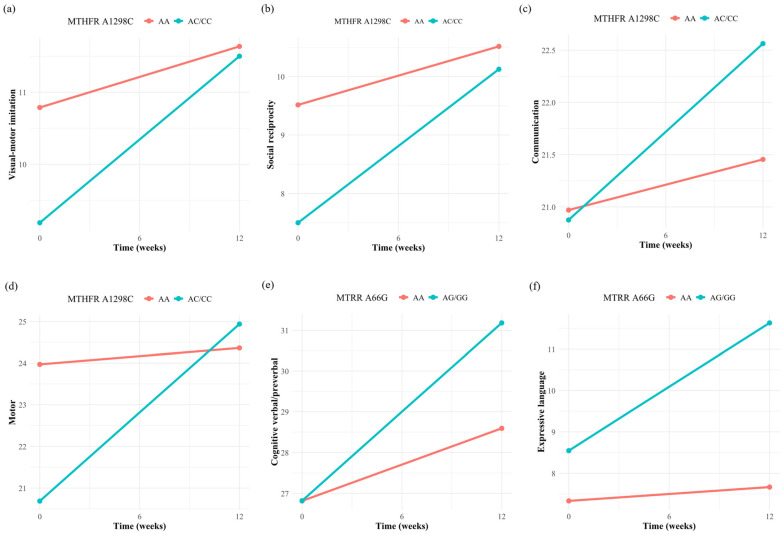
Changes in PEP-3 scores by MTHFR A1298C and MTRR A66G genotypes following 12 weeks of folinic acid intervention. Children with the MTHFR A1298C variant (AC/CC) showed greater improvements in the visual-motor imitation (**a**), social reciprocity (**b**), the composite score of communication (**c**) and motor (**d**) compared to the wild-type. Children with the MTRR A66G variant (AG/GG) also showed greater improvement in cognitive verbal/preverbal (**e**) and expressive language (**f**) compared to the wild type.

The results of the linear mixed model adjusted for all covariates showed that children carrying the *MTHFR* A1298C variant (AC/CC) showed greater improvements across several domains compared to those with the wild-type genotype, including an additional increase of 1.46 points in visual-motor imitation, 1.63 points in social reciprocity, 1.20 points in the composite score of communication, and 3.86 points in the composite score of motor. Notably, the baseline scores for visual-motor imitation, social reciprocity, and the composite score of communication and motor were initially lower in the mutant group than in the wild type. After three months of intervention, the composite score of communication and motor in the mutant group surpassed those of the wild type (Table 3 and Figure 2a–d). Additionally, children carrying the *MTRR* A66G variant (AG/GG) also showed greater improvements in cognitive verbal/preverbal and expressive language compared with children with the wild-type genotype, with an additional increase of 2.59 points and 2.76 points, respectively (Table 3 and Figure 2e,f).

### 3.4. Association of Folate Metabolism Gene Polymorphism Combination with Efficacy

Compared with the combined wild-type of *MTHFR* C677T and *MTHFR* A1298C, participants with *MTHFR* 677CC and *MTHFR* 1298 AC/CC genotype showed a significant positive effect on the composites of communication (β = 2.38, 95% CI: 0.24, 4.51; *p* = 0.030) and motor (β = 6.33, 95% CI: 1.77, 10.89; *p* = 0.008). Participants with *MTHFR* 677 CT/TT and *MTHFR* 1298AA genotypes showed a significant positive effect on characteristic verbal behaviors (β = 2.45, 95% CI: 0.45, 4.45; *p* = 0.018) and the composite scores of maladaptive behaviors (β = 3.96, 95% CI: 0.39, 7.52; *p* = 0.031). Moreover, participants with *MTHFR* 677 CT/TT and *MTHFR* 1298 AC/CC genotypes also showed a significant positive effect on the composite score of motor (β = 6.07, 95% CI: 0.17, 11.97; *p* = 0.044) and maladaptive behaviors (β = 6.52, 95% CI: 0.99, 12.06; *p* = 0.022). Similarly, participants with *MTHFR* 1298 AC/CC and *MTRR* 66AA genotypes showed a significant positive effect on the fine motor (β = 2.54, 95% CI: 0.10, 4.97, *p* = 0.042) and the composite score of motor (β = 5.28, 95% CI: 1.27, 9.29; *p* = 0.012) than those with the combined wild-type of *MTHFR* A1298C and *MTRR* A66G. Additionally, participants with *MTR* 2756 AA and *MTRR* 66AG/GG genotypes showed a significant positive effect on expressive language (β = 2.81, 95% CI: 0.05, 5.56; *p* = 0.046) compared to those with the combined wild-type of *MTR* A2756G and *MTRR* A66G (Supplementary Materials, Table S8).

## 4. Discussion

This study demonstrated that a 12-week high-dose folinic acid intervention can improve social reciprocity in children with ASD. Moreover, the folinic acid supplement in this trial was well tolerated among children with ASD, and no significant adverse reactions were reported during the observation period. These findings regarding the potential benefits of folinic acid interventions in children with ASD are consistent with results from several studies conducted in other countries [11,16,36]. For example, randomized controlled trials conducted in the United States [11] and France [16] have demonstrated that high-dose folinic acid can significantly improve the severity of autism symptoms. Similarly, an open-label trial conducted in Singapore reported that children with ASD had greater improvements in social interaction and expressive language after folinic acid supplementation [37]. Folinic acid has also shown efficacy as an adjuvant therapy alongside other medications like risperidone or methylcobalamin, leading to improvements in symptoms such as inappropriate speech, hyperactivity, and adaptive behavior [14,15]. Moreover, folinic acid treatment for ASD offers significant cost savings compared to speech therapy, with equivalent benefits at a lower cost and a low frequency of adverse effects [6]. However, an open-label study did not observe significant improvements after high-dose folinic acid supplementation [38]. This difference may be related to the small sample size (n = 12) and the relatively older age range of the ASD subjects (13-19 years old). Studies suggested that younger children with ASD showed greater improvement in core symptoms following folinic acid supplementation compared to older children, possibly due to greater neuroplasticity during early developmental stages [39]. Future large-scale longitudinal studies will be necessary to determine whether there is a critical developmental window during which folinic acid supplementation may yield maximal therapeutic benefit.

The results of this study also found that children carrying the *MTHFR* A1298C mutation (AC/CC genotype) exhibited significantly greater improvements in multiple developmental areas, including visual-motor imitation, social reciprocity, communication, and motor skills than the wild-type genotype following folinic acid intervention. Moreover, children carrying the *MTRR* A66G mutation (AG/GG genotype) showed greater improvements in cognitive verbal/preverbal and expressive language skills than the wild-type genotype. The potential mechanisms underlying the better therapeutic response to folinic acid intervention in patients carrying the *MTHFR* A1298C and *MTRR* A66G variants may involve the following biological pathways. Both the *MTHFR* A1298C and *MTRR* A66G mutations can reduce the activity of their encoded enzymes, resulting in impaired folate metabolism, elevated homocysteine levels, and DNA hypomethylation [22,23,24]. These can disrupt neurodevelopment by impairing nucleotide synthesis, increasing oxidative stress and neuroinflammation, and altering gene expression regulation, which are also known pathological mechanisms underlying ASD [40]. High-dose folinic acid intervention can significantly increase 5,10-methylenetetrahydrofolate concentrations, which can increase 5-methyltetrahydrofolate levels and enhance folate metabolism in the presence of impaired MTHFR enzyme activity. Additionally, research suggests that folate levels may significantly influence the stability of the polymorphic enzyme [25]. For instance, at higher intracellular folate levels, the folate molecule can stabilize the variant MTHFR protein by maintaining its three-dimensional structure in the appropriate and fully functional form, thereby mitigating the thermolabile form and offsetting the reduction in enzyme activity [41]. In this study, the mutation frequencies of *MTHFR* C677T, *MTHFR* A1298C, *MTR* A2756G, and *MTRR* A66G among ASD patients were 59.18%, 32.65%, 24.49%, and 44.90%, respectively. These are similar to previously published studies in the Chinese populations [42,43]. Notably, the distribution of these genotypes in the Chinese population is different from that in other countries. The prevalence of *MTHFR* C677T and *MTRR* A66G mutations is significantly higher in the Chinese population, while *MTHFR* A1298C and *MTR* A2756G mutations are more frequent in other countries [19,44]. These differences may result in distinct baseline folate metabolic profiles and thus variable responses to folinic acid supplementation.

This study suggests that specific combinations of folate metabolism gene polymorphisms show better therapeutic effects. For example, compared with the combined wild-type genotype, children with *MTHFR* 677 CC and 1298 AC/CC genotypes showed significantly greater improvements in the composite scores of communications and motor, while children with *MTHFR* 677 CT/TT and 1298 AA or 1298 AC/CC genotypes showed significantly greater improvements in characteristic verbal behaviors, the composite scores of motor and maladaptive behaviors. This may be due to the synergistic effects between folate metabolism gene polymorphisms, which collectively increase the risk of folate metabolism disorders, elevated homocysteine levels, and DNA hypomethylation to varying degrees, thereby leading to different responses to folic acid supplementation [21,25]. Studies have shown that individuals with compound heterozygous mutations of *MTHFR* C677T and A1298C exhibit significantly higher plasma homocysteine levels, lower plasma folate levels, and DNA hypomethylation than those with only a single mutation [25]. Individuals with the *MTHFR* 677TT and 1298AA genotypes manifest the highest homocysteine, lowest plasma folate levels, and lowest DNA methylation [23]. Therefore, high-dose folinic acid intervention may promote better neurodevelopmental outcomes by alleviating abnormal folate metabolism caused by single or combined mutations in folate metabolism genes.

The study has several strengths. First, to our knowledge, it is the first to evaluate the safety and efficacy of high-dose folinic acid intervention among children with ASD in China, contributing valuable insights to a population that has been underrepresented in previous research. Second, the study assessed the association of single and combined folate metabolism gene polymorphisms with treatment response, providing a more comprehensive understanding of the relationship between genetic factors and efficacy. These results suggest that children carrying specific folate metabolism genotypes may benefit more from high-dose folinic acid supplementation, which supports the rationale of genotype-guided therapeutic strategies. As genetic testing becomes more accessible and affordable, incorporating genotyping into routine clinical practice is becoming an increasingly feasible strategy, which can provide more precise and effective interventions for ASD. Future studies should validate the associations between these genotypes and treatment responses in larger cohorts to inform evidence-based clinical decision making. Third, the use of a validated developmental assessment tool (PEP-3) allowed for a comprehensive evaluation of the efficacy of folinic acid intervention. However, this study has several limitations. Firstly, the open-label design may introduce bias. To reduce bias, objective PEP-3 assessments were preferred over parent-reported measures in this study. The evaluation was performed by two independent professionals who were blinded to group assignment. Secondly, the small sample size for some genotypes in the intervention group and the lack of genotyping data in the control group may weaken the strength of causal inference. Moreover, this study did not perform statistical corrections for multiple testing, which may result in false-positive results. Future studies should employ a double-blind, placebo-controlled design, increase sample size, and conduct long-term follow-up to strengthen the reliability and generalizability of the findings.

## 5. Conclusions

In conclusion, high-dose folinic acid can improve social reciprocity in children with ASD, and its efficacy is associated with folate metabolism gene polymorphisms. High-dose folinic acid intervention may promote better neurodevelopmental outcomes by alleviating folate metabolism abnormalities caused by single or combined mutations in folate metabolism genes. Future research should focus on the relationship between genetic factors and treatment response, aiming to provide more effective therapeutic interventions for children with ASD through precision medicine strategies.

## Figures and Tables

**Table 1:** Baseline demographic and clinical characteristics.

Characteristic	Total (*n* = 76)	Control Group (*n* = 27)	Intervention Group (*n* = 49)	*p*
Gender				0.745
Female	12 (15.8%)	5 (18.5%)	7 (14.3%)	
Male	64 (84.2%)	22 (81.5%)	42 (85.7%)	
Age at enrollment, months	51.28 (10.90)	51.30 (12.94)	51.27 (9.75)	0.991
Ethnicity				0.650
Han	71 (93.4%)	26 (96.3%)	45 (91.8%)	
Other	5 (6.6%)	1 (3.7%)	4 (8.2%)	
Preterm birth				0.999
No	69 (90.8%)	25 (92.6%)	44 (89.8%)	
Yes	7 (9.2%)	2 (7.4%)	5 (10.2%)	
Birth weight, kg	3.28 (0.43)	3.42 (0.43)	3.21 (0.42)	0.041
Mode of delivery				0.328
Vaginal delivery	46 (60.5%)	14 (51.9%)	32 (65.3%)	
Cesarean section	30 (39.5%)	13 (48.1%)	17 (34.7%)	
Paternal age at birth, years	31.51 (5.57)	31.19 (6.14)	31.69 (5.28)	0.718
Maternal age at birth, years	29.33 (4.89)	28.89 (5.34)	29.57 (4.65)	0.580
Father’s education level				0.355
Less than high school	13 (17.1%)	7 (25.9%)	6 (12.2%)	
High school	15 (19.7%)	5 (18.5%)	10 (20.4%)	
College degree or higher	48 (63.2%)	15 (55.6%)	33 (67.3%)	
Mother’s education level				0.027
Less than high school	11 (14.5%)	8 (29.6%)	3 (6.1%)	
High school	25 (32.9%)	8 (29.6%)	17 (34.7%)	
College degree or higher	40 (52.6%)	11 (40.7%)	29 (59.2%)	
ABC scores	59.42 (14.18)	60.44 (14.04)	58.86 (14.36)	0.642
CARS scores	36.24 (4.00)	35.96 (4.59)	36.39 (3.68)	0.681

Note: Continuous variables are presented as mean (SD) and compared using the *t*-test; categorical variables are shown as frequencies (%) and compared using Fisher’s exact test; abbreviations: ABC, Autism Behavior Checklist; CARS, Childhood Autism Rating Scale.

**Table 2:** Comparison of baseline and 12-week scores and score changes between intervention and control groups.

Characteristic	Control Group (*n* = 27)			Intervention Group (*n* = 49)			Between-Group Difference in Change	
	Baseline	12-Weeks	Change from Baseline	Baseline	12-Weeks	Change from Baseline	Mean Difference (95% CI)	*p*
Subtest								
Cognitive verbal/preverbal	29.07 (15.11)	33.96 (15.72)	4.89 (7.03)	26.82 (12.72)	29.76 (13.30)	2.94 (4.36)	−1.95 (−4.55, 0.65)	0.140
Expressive language	10.89 (12.08)	14.00 (13.09)	3.11 (4.39)	7.88 (9.97)	9.45 (11.97)	1.57 (3.76)	−1.54 (−3.45, 0.37)	0.112
Receptive language	14.52 (11.05)	18.89 (10.92)	4.37 (5.43)	13.22 (9.67)	15.65 (11.00)	2.43 (3.61)	−1.94 (−4.01, 0.13)	0.066
Fine motor	27.22 (7.24)	28.74 (7.00)	1.52 (3.95)	26.65 (6.57)	28.41 (6.49)	1.76 (2.77)	0.24 (−1.31, 1.78)	0.761
Gross motor	22.41 (5.72)	23.96 (5.85)	1.56 (2.81)	23.06 (5.78)	24.49 (5.01)	1.43 (3.40)	−0.13 (−1.66, 1.40)	0.869
Visual-motor imitation	11.00 (4.81)	12.22 (4.53)	1.22 (2.03)	10.27 (4.73)	11.59 (4.61)	1.33 (2.42)	0.10 (−0.99, 1.20)	0.850
Affective expression	10.33 (3.46)	10.26 (2.85)	−0.07 (2.06)	9.61 (2.56)	9.92 (2.57)	0.31 (1.88)	0.38 (−0.55, 1.31)	0.418
Social reciprocity	10.19 (3.56)	10.52 (3.18)	0.33 (1.71)	8.86 (3.07)	10.39 (3.43)	1.53 (1.98)	1.20 (0.29, 2.10)	0.010
Characteristic motor behaviors	17.15 (4.15)	17.70 (4.67)	0.56 (3.09)	15.94 (4.45)	15.73 (4.64)	−0.20 (3.24)	−0.76 (−2.28, 0.76)	0.324
Characteristic verbal behaviors	6.56 (5.92)	7.89 (5.70)	1.33 (2.57)	4.29 (4.50)	5.10 (5.19)	0.82 (2.19)	−0.52 (−1.63, 0.6)	0.357
Composites								
Communication	23.15 (8.27)	23.93 (9.38)	0.78 (4.29)	20.94 (7.38)	21.82 (7.70)	0.88 (1.91)	0.10 (−1.32, 1.52)	0.889
Motor	24.74 (7.82)	24.78 (9.87)	0.04 (6.30)	22.90 (8.98)	24.55 (8.91)	1.65 (4.53)	1.62 (−0.88, 4.11)	0.200
Maladaptive behaviors	27.85 (9.13)	27.44 (8.87)	−0.41 (5.57)	24.39 (7.57)	25.14 (7.82)	0.76 (3.70)	1.16 (−0.96, 3.29)	0.279
Caregiver report								
Problem behavior	8.70 (3.34)	9.22 (4.08)	0.52 (2.31)	8.63 (3.57)	9.15 (4.06)	0.50 (2.90)	−0.02 (−1.31, 1.28)	0.977
Personal self-care	16.41 (4.75)	17.30 (4.43)	0.89 (2.14)	16.08 (4.80)	17.25 (4.52)	1.33 (3.39)	0.44 (−1.00, 1.89)	0.541
Adaptive behaviors	17.33 (5.72)	18.41 (5.49)	1.07 (3.30)	15.71 (6.18)	17.23 (6.84)	1.52 (3.41)	0.45 (−1.17, 2.07)	0.584

**Table 3:** The association between single folate metabolism gene polymorphisms and intervention efficacy.

Variables	*MTHFR* A1298C		*MTHFR* C677T		*MTR* A2756G		*MTRR* A66G	
	Estimate (95% CI)	*p*	Estimate (95% CI)	*p*	Estimate (95% CI)	*p*	Estimate (95% CI)	*p*
Subtest								
Cognitive verbal/preverbal	1.20 (−1.22, 3.63)	0.370	−1.20 (−3.51, 1.10)	0.348	−2.02 (−4.63, 0.60)	0.166	2.59 (0.37, 4.80)	0.038
Expressive language	0.73 (−1.25, 2.71)	0.514	−0.22 (−2.11, 1.68)	0.839	−1.86 (−3.99, 0.26)	0.124	2.76 (1.02, 4.50)	0.006
Receptive language	0.76 (−1.16, 2.67)	0.485	0.64 (−1.19, 2.47)	0.536	−0.68 (−2.77, 1.41)	0.566	0.38 (−1.43, 2.18)	0.711
Fine motor	1.01 (−0.42, 2.45)	0.214	−1.09 (−2.46, 0.28)	0.161	−1.11 (−2.67, 0.45)	0.211	1.35 (0.01, 2.70)	0.078
Gross motor	0.85 (−0.91, 2.60)	0.393	−0.71 (−2.39, 0.97)	0.454	0.98 (−0.94, 2.89)	0.367	0.62 (−1.04, 2.29)	0.506
Visual-motor imitation	1.46 (0.29, 2.64)	0.030	−0.04 (−1.20, 1.12)	0.952	0.34 (−0.98, 1.66)	0.649	0.40 (−0.74, 1.54)	0.538
Affective expression	0.10 (−0.91, 1.11)	0.858	0.52 (−0.44, 1.48)	0.342	0.48 (−0.63, 1.59)	0.446	−0.14 (−1.10, 0.82)	0.792
Social reciprocity	1.63 (0.66, 2.59)	0.004	−0.03 (−1.01, 0.95)	0.953	−0.15 (−1.27, 0.97)	0.811	−0.39 (−1.35, 0.58)	0.480
Characteristic motor behaviors	0.03 (−1.68, 1.73)	0.980	0.50 (−1.13, 2.13)	0.587	0.93 (−0.92, 2.78)	0.373	−0.62 (−2.22, 0.98)	0.492
Characteristic verbal behaviors	−0.93 (−2.12, 0.25)	0.163	1.13 (0.01, 2.24)	0.076	0.02 (−1.31, 1.36)	0.976	0.09 (−1.07, 1.24)	0.893
Composites								
Communication	1.20 (0.20, 2.21)	0.037	−0.12 (−1.12, 0.88)	0.828	−0.72 (−1.85, 0.41)	0.261	0.39 (−0.60, 1.37)	0.486
Motor	3.86 (1.62, 6.09)	0.003	−0.67 (−2.97, 1.63)	0.606	0.02 (−2.62, 2.66)	0.990	0.13 (−2.14, 2.41)	0.917
Maladaptive behaviors	1.57 (−0.48, 3.62)	0.166	1.70 (−0.20, 3.59)	0.115	1.32 (−0.92, 3.55)	0.288	−1.21 (−3.13, 0.72)	0.261
Caregiver report								
Problem behavior	0.10 (−1.44, 1.65)	0.906	0.69 (−0.78, 2.15)	0.409	−0.77 (−2.46, 0.92)	0.420	−0.40 (−1.86, 1.07)	0.631
Personal self-care	−0.39 (−2.15, 1.37)	0.696	−0.30 (−1.99, 1.40)	0.756	0.57 (−1.35, 2.49)	0.601	−1.04 (−2.70, 0.63)	0.272
Adaptive behaviors	1.15 (−0.70, 3.00)	0.273	−0.67 (−2.45, 1.11)	0.508	−1.08 (−3.08, 0.92)	0.342	1.18 (−0.57, 2.93)	0.235

Note: Adjusted for baseline scores of each outcome variable, age at enrollment, ethnicity, preterm birth, birth weight, mode of delivery, paternal age at birth, maternal age at birth, father’s education, father’s education level, scores of the Autism Behavior Checklist (ABC), and Childhood Autism Rating Scale (CARS).

## Data Availability

Data described in the manuscript, code book, and software will be made available via e-mail request to the corresponding author.

## Supplementary Materials

The following supporting information can be downloaded at: https://www.mdpi.com/article/10.3390/nu17091602/s1, Table S1: PCR amplification reaction system; Table S2: PCR procedure settings; Table S3: Primer sequences and PCR product lengths for genotyping of folate metabolism genes; Table S4: Summary of genotyping results for MTHFR C677T, MTHFR A1298C, MTR A2756G, and MTRR A66G polymorphisms; Table S5: Hardy-Weinberg equilibrium test results for folate metabolism gene polymorphisms (n = 49); Table S6: Baseline characteristics of children with ASD in the intervention group; Table S7: Comparison of baseline scores between wild-type and mutant genotypes for each variable; Table S8: The combined effects of folate metabolism gene polymorphisms on intervention response.

## Abbreviations

List of Abbreviations in alphabetical order.

ABC	Autism Behavior Checklist
ASD	Autism Spectrum Disorder
CARS	Childhood Autism Rating Scale
*MTHFR*	Methylenetetrahydrofolate reductase
*MTR*	Methionine synthase
*MTRR*	Methionine synthase reductase
PEP-3	Psycho-Educational Profile, Third Edition
PCR	Polymerase chain reaction
RCT	Randomized controlled trial
